# The proteomics of the giant cedratvirus particles reveals unique and shared features with pitho-like viruses

**DOI:** 10.1128/jvi.00830-25

**Published:** 2025-12-04

**Authors:** Talita B. Machado, Talita D. Melo-Hanchuk, Bruna L. de Azevedo, Isabella L. M. de Aquino, Juliana R. Cortines, Rafael E. Marques, Jônatas S. Abrahão

**Affiliations:** 1 Laboratório de Vírus, Departamento de Microbiologia, Instituto de Ciências Biológicas, Universidade Federal de Minas Gerais (UFMG) 549116 , Belo Horizonte, Minas Gerais, Brazil; 2 Brazilian Biosciences National Laboratory (LNBio), Brazilian Center for Research in Energy and Materials (CNPEM) 215006, Campinas, Brazil; 3 Centro de Tecnologia de Vacinas (CT Vacinas)/BH-Tec, Universidade Federal de Minas Gerais (UFMG) 28114, Belo Horizonte, Minas Gerais, Brazil; 4 Departamento de Virologia, Instituto de Microbiologia Paulo de Góes, Universidade Federal do Rio de Janeiro 28125, Rio de Janeiro, Rio de Janeiro, Brazil; Michigan State University, East Lansing, Michigan, USA

**Keywords:** proteomics, giant virus, *Pithoviridae*, cedratvirus pambiensis

## Abstract

Cedratvirus is a giant virus belonging to the order *Pimascovirales*, a group that includes other viruses such as pithoviruses and orpheoviruses. Cedratviruses infect *Acanthamoeba castellanii* and have a genome of ~600 kb, encoding ~800 predicted proteins. In this study, we performed, for the first time to our knowledge, a proteomic analysis of purified cedratvirus particles, identifying 266 proteins associated with the virus. These proteins were involved in a variety of cellular processes, including signal transduction regulation, transcription, RNA processing, DNA replication, recombination, and repair, among others. Additionally, we identified in cedratvirus particles 172 host-associated proteins. A comparative analysis of the cedratvirus proteome with that of pithovirus sibericum revealed that 89 proteins are shared between the two viruses, while 96 proteins are unique to cedratvirus. These findings provide the first comprehensive proteomic characterization of a cedratvirus, advancing our understanding of its molecular biology and highlighting both conserved and unique features within the pimascoviruses, which may inform future studies on viral evolution and host-virus interactions.

**IMPORTANCE:**

There are still only a few studies on the proteomics of giant viruses. Research published to date has revealed the presence of several proteins involved in processes such as transcription (including RNA polymerase subunits, helicases, and transcription factors), DNA topology regulation (e.g., topoisomerases), and metabolic pathways (e.g., enzymes that help the virus cope with host oxidative stress). Proteomic studies of giant viruses are important, as understanding the role of these proteins can provide information about viral biology, host-virus interactions, and viral evolution. Here, we present, for the first time to our knowledge, a proteomic analysis of a cedratvirus, highlighting unique and shared features with pithovirus, a relative.

## INTRODUCTION

The discovery of giant viruses has generated significant interest in the scientific community due to their unprecedented particle size and the complexity of their genomes (mimivirus, Marseillevirus, Pandoravirus, pithovirus, Faustovirus, mollivirus, cedratvirus, kaumoebavirus, Pacmanvirus, Orpheovirus, and Medusavirus) (1–11). In 2014, the isolation of Pithovirus sibericum captured widespread attention, particularly because of its large particle size, measuring approximately 1,300 nm in length (4). Similar to pithovirus, its relatives cedratviruses and orpheoviruses feature large viral particles, ranging in size from 900 to 1,400 nm (7, 10). Four years later, the discovery of Tupanvirus, which has a similarly large particle size ranging from 1,200 to 2,300 µm, further expanded our understanding of these exceptional viruses. In addition to their impressive size, Tupanvirus revealed a sophisticated translational apparatus, underscoring the complexity of giant viruses (12). Since these viruses possess numerous genes that are not only novel to the virosphere but also unique in their functions, studying the proteome of giant viruses’ particles has become essential to understand their biology.

While proteomic studies on giant viruses remain relatively limited, these investigations are essential for expanding our understanding of the virus-host interactions, as well as the ecological roles these viruses play in nature. For instance, proteomic analyses of giant viruses have revealed the presence of proteins involved in transcription and DNA repair, suggesting that these viruses are capable of initiating genome replication and transcription with a certain degree of autonomy shortly after infecting their host (2, 3, 12–15). This ability to manage essential metabolic processes is a defining characteristic of many giant viruses, setting them apart from smaller, more traditional viruses. A particularly fascinating feature of giant viruses is the high prevalence of ORFans (open reading frames with no detectable homologs), which are genes encoding proteins with unknown functions. For example, in the genome of mimivirus (APMV), ORFans account for approximately 67% of the viral genome (16), while in Pandoravirus salinus, they make up about 93% (3). These ORFans, which are often identified in proteomic analyses, represent a significant mystery in the field. Since their functions remain unknown, it is challenging to predict their roles or importance in the viral life cycle. Proteomic studies are therefore important in confirming the existence of ORFans predicted by bioinformatics, helping to establish their presence in the viral particle and guiding future functional investigations.

In this study, we focus on cedratvirus pambiensis, a giant virus with a genome of 623,564 base pairs and 842 predicted proteins (17). This is the first proteomic analysis of cedratvirus particles according to our knowledge. We found proteins involved in a variety of processes, including signal transduction, transcription and RNA processing, DNA replication, recombination, and repair, as well as various metabolic pathways. Additionally, we performed a comparative analysis with cedratvirus relative, pithovirus sibericum, highlighting the conserved and possibly important roles of certain viral proteins. Taken together, our data contribute to our understanding of the proteomic landscape of cedratvirus pambiensis and its evolutionary relationship with other pitho-like viruses.

## RESULTS

### Proteomic characterization of edratvirus pambiensis

**Fig 1:**
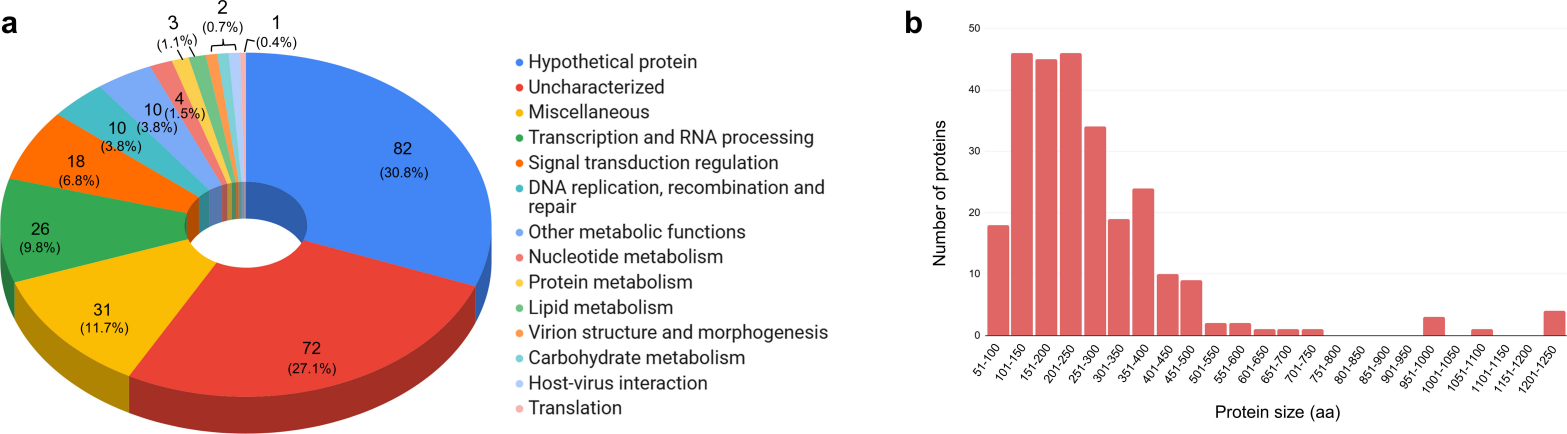
Proteomic characterization of cedratvirus pambiensis. (**a**) Functional category distribution of proteins identified in the cedratvirus pambiensis proteome. (**b**) Distribution of proteins according to size (aa).

In this study, we conducted a comprehensive proteomic analysis of purified cedratvirus pambiensis particles, resulting in the identification of 266 proteins (Table 1), which accounts for approximately 31.6% of the proteins predicted by Machado et al. (17). The identified proteins are involved in a wide array of essential cellular processes, highlighting the complexity of this giant virus. Functional characterization of these proteins revealed their involvement in key viral and host-associated mechanisms, including signal transduction regulation, DNA replication, recombination and repair, transcription, and RNA processing (Fig. 1a; Table 1; see Table S1 at https://www.giantviruses.com/sup-material-of-papers/sup-material-the-proteomics-of-the-giant-cedratvirus-particles-reveals-unique-and-shared-features-with-pitho-like-viruses). Notably, the presence of proteins associated with DNA repair and replication suggests that the virus may be capable of managing key processes soon after infecting the host cell, similar to other giant viruses. Additionally, the identification of proteins involved in transcription and RNA processing suggests that cedratvirus pambiensis may also regulate its own gene expression, which is consistent with electron microscopy findings indicating that the replication cycle of cedratvirus pambiensis takes place in the amoebal cytoplasm, probably without requiring the amoeba’s transcription machinery. Although cedratvirus pambiensis has 76 predicted ORFans (9.03% of its genome), none of them were detected in our proteome analyses.

**TABLE 1:** Proteins identified in cedratvirus pambiensis particles and their functions inferred from BLASTp best hits

Gene ID	Function	Mol. weight (kDa)	Score	LFQ media	Unique peptides	Sequence coverage (%)
30	Serine/Threonine protein kinase [cedratvirus Ce7-1]	48.493	323.31	23,05845451	8	25.2
50	DNA-directed RNA polymerase subunit RPB2 [Brazilian cedratvirus IHUMI]	136.77	323.31	25,54757373	13	49
51	Endoribonuclease L-PSP/chorismate mutase-like protein [Brazilian cedratvirus IHUMI]	19.794	323.31	28,92235247	4	50.8
169	Uncharacterized protein Ce0701_0016 [cedratvirus Ce7-1]	37.864	323.31	27,48398081	15	45.2
181	DNA-directed RNA polymerase subunit [cedratvirus Ce7-1]	107.79	323.31	26,93042056	29	69
183	Uncharacterized protein Ce0701_0029 [cedratvirus Ce7-1]	31.819	323.31	30,96556981	7	84.1
191	DNA-directed RNA polymerase subunit RPB1 [Brazilian cedratvirus IHUMI]	107.47	323.31	24,62430064	23	60.9
196	Uncharacterized protein Ce0701_0032 [cedratvirus Ce7-1]	29.706	323.31	26,80596161	7	43.8
203	Hypothetical protein BRZCDTV_384 [Brazilian cedratvirus IHUMI]	60.725	323.31	32,34989802	19	68.3
205	Class three lipase [Brazilian cedratvirus IHUMI]	31.591	323.31	26,01503944	7	47.9
239	Class three lipase [Brazilian cedratvirus IHUMI]	31.913	323.31	27,04893557	7	47.3
267	Nucleotidyl transferase [cedratvirus Ce7-1]	49.593	323.31	25,30985133	12	38.2
275	Hypothetical protein BRZCDTV_349 [Brazilian cedratvirus IHUMI]	12.478	323.31	30,38724327	9	74.3
282	Hypothetical protein BRZCDTV_349 [Brazilian cedratvirus IHUMI]	12.31	323.31	29,76369603	11	89.4
284	Hypothetical protein Cplu_202 [cedratvirus plubellavi]	34.845	323.31	29,55129751	11	43.8
297	Hypothetical protein BRZCDTV_334 [Brazilian cedratvirus IHUMI]	40.504	323.31	29,00968424	26	66.1
306	Hypothetical protein BRZCDTV_336 [Brazilian cedratvirus IHUMI]	16.107	323.31	28,51016935	8	91.5
319	Protein containing kinase domain [Brazilian cedratvirus IHUMI]	64.775	323.31	30,79834239	1	56
328	Hypothetical protein BRZCDTV_316 [Brazilian cedratvirus IHUMI]	35.85	323.31	26,55948957	12	59.7
377	Transmembrane domain-containing protein [cedratvirus Ce7-1]	13.202	323.31	27,28659248	3	37.8
385	Hypothetical protein BRZCDTV_286 [Brazilian cedratvirus IHUMI]	17.351	323.31	29,38215192	9	82.3
406	VV D6-like helicase [Brazilian cedratvirus IHUMI]	124.92	323.31	28,32836914	61	59.9
415	Uncharacterized protein Ce0701_0419 [cedratvirus Ce7-1	30.876	323.31	30,36526362	21	82.8
450	Uncharacterized protein Ce0701_0228 [cedratvirus Ce7-1]	37.397	323.31	31,78368187	7	43.8
476	Uncharacterized protein Ce0201_0148 [cedratvirus Ce2-1]	44.949	323.31	24,75685755	8	58.5
482	Hypothetical protein BRZCDTV_230 [Brazilian cedratvirus IHUMI]	36.597	323.31	27,13981883	13	70.1
483	Uncharacterized protein Ce0701_0243 [cedratvirus Ce7-1]	44.913	323.31	26,5209287	13	69
493	DNA-directed RNA polymerase RPB1 (Domain 5) [cedratvirus Ce7-1]	78.429	323.31	26,76166789	18	41.2
523	Uncharacterized protein Ce0701_0188 [cedratvirus Ce7-1]	33.986	323.31	24,6823171	12	67.6
537	Protein containing kinase domain [Brazilian cedratvirus IHUMI]	41.604	323.31	27,89632352	21	52.2
563	Hypothetical protein [cedratvirus kamchatka]	24.295	323.31	32,43375905	22	60.1
597	Hypothetical protein BRZCDTV_159 [Brazilian cedratvirus IHUMI]	13.058	323.31	30,77889061	10	53.8
608	Hypothetical protein BRZCDTV_159 [Brazilian cedratvirus IHUMI]	39.75	323.31	31,35135206	15	66.2
621	Hypothetical protein BRZCDTV_149 [Brazilian cedratvirus IHUMI]	34.348	323.31	32,57397079	27	77.2
636	7-methylguanosine mRNA capping enzyme [Brazilian cedratvirus IHUMI]	111.53	323.31	27,09770902	62	57.6
651	HMG box domain-containing protein [cedratvirus Ce2-1]	25.722	323.31	31,88154093	18	78.4
665	Hypothetical protein BRZCDTV_123 [Brazilian cedratvirus IHUMI]	23.978	323.31	32,87328593	24	88.6
666	VETF-like early transcription factor large subunit [cedratvirus Ce7-1]	138.81	323.31	26,74043338	35	51.4
677	Uncharacterized protein Ce0701_0166 [cedratvirus Ce7-1]	30.554	323.31	27,47323354	19	62.8
678	Hypothetical protein BRZCDTV_113 [Brazilian cedratvirus IHUMI]	25.857	323.31	26,75631332	15	56.3
679	Ankyrin repeat-containing protein [cedratvirus Ce7-1]	34.176	323.31	27,36411985	25	74.5
684	Ankyrin repeat-containing protein [cedratvirus Ce2-1]	51.637	323.31	27,04169782	33	66.6
706	Serine/Threonine protein kinase [Brazilian cedratvirus IHUMI]	53.856	323.31	27,1447614	18	51.6
725	Lipocalin/cytosolic fatty-acid binding domain-containing protein [cedratvirus Ce7-1]	19.047	323.31	29,46610578	3	71.4
778	Uncharacterized protein Ce0701_0127 [cedratvirus Ce7-1]	43.439	323.31	26,03309123	22	57.8
789	Hypothetical protein BRZCDTV_27 [Brazilian cedratvirus IHUMI]	40.755	323.31	28,72144127	15	43.1
807	F-box domain-containing protein [Brazilian cedratvirus IHUMI]	23.293	323.31	28,89256477	9	61.3
243	Ankyrin repeat-containing protein [cedratvirus Ce7-1]	42.678	314.45	26,34749794	12	58
572	Hypothetical protein BRZCDTV_169 [Brazilian cedratvirus IHUMI]	54.627	311.51	26,91512489	22	48.3
780	F-box domain-containing protein [cedratvirus Ce2-1]	23.952	305.22	27,82586288	10	61.5
338	Hypothetical protein BRZCDTV_309 [Brazilian cedratvirus IHUMI]	69.598	290.16	28,44070244	2	73.8
514	Uncharacterized protein Ce0701_0188 [cedratvirus Ce7-1]	34.794	286.85	27,34329224	12	65.1
542	Hypothetical protein BRZCDTV_180 [Brazilian cedratvirus IHUMI]	48.826	284.44	26,97695796	8	46.8
582	Hypothetical protein BRZCDTV_169 [Brazilian cedratvirus IHUMI]	55.841	278.72	25,67181778	19	48.8
104	Hypothetical protein BRZCDTV_453 [Brazilian cedratvirus IHUMI]	33.216	277.02	26,83143552	18	52.7
226	Hypothetical protein Cbor_269 [cedratvirus borely]	43.073	271.66	26,43916893	25	68
671	5'−3' exoribonuclease [Brazilian cedratvirus IHUMI]	37.669	265.69	284.935.201	3	44.7
817	F-box domain-containing protein [cedratvirus Ce7-1]	23.31	264.55	26,57676061	6	60.3
255	Uncharacterized protein Ce0701_0065 [cedratvirus Ce7-1]	42.85	261.67	27,12876002	21	60.4
652	VETF-like early transcription factor large subunit [Cedratvirus Ce7-1]	138.89	258.96	25,65801048	33	50.6
252	Uncharacterized protein Ce0701_0057 [cedratvirus Ce7-1]	22.185	253.35	26,81634903	7	49.5
602	Hypothetical protein BRZCDTV_159 [Brazilian cedratvirus IHUMI]	28.896	253.27	30,74014473	9	64.5
363	Transmembrane domain-containing protein [cedratvirus Ce7-1]	29.582	250.16	29,49365107	11	46.1
325	Uncharacterized protein Ce0701_0269 [cedratvirus Ce7-1]	22.066	248.42	30,79998016	11	69.7
168	F-box domain-containing protein [cedratvirus Ce7-1]	26.953	247.75	26,94149272	19	82.7
840	Hypothetical protein Cbor_452 [cedratvirus borely]	19.809	245.33	27,70615768	16	67.1
186	Uncharacterized protein Ce0701_0032 [cedratvirus Ce7-1]	22	244.98	27,12283007	5	48.3
704	Cyclin dependent kinase 2 [Brazilian cedratvirus IHUMI]	52.175	240.5	26,61201286	19	47
331	Hypothetical protein BRZCDTV_316 [Brazilian cedratvirus IHUMI]	36.037	234.09	25,6137619	7	53
784	Hypothetical protein BRZCDTV_32 [Brazilian cedratvirus IHUMI]	10.174	232.94	27,99599457	10	91.4
223	F-box domain-containing protein [cedratvirus Ce7-1]	20.963	228.87	25,25804647	10	49.2
295	Hypothetical protein BRZCDTV_336 [Brazilian cedratvirus IHUMI]	16.158	225.28	28,81258265	6	85.9
556	Hypothetical protein BRZCDTV_176 [Brazilian cedratvirus IHUMI]	11.751	219.53	24,75868479	3	36.6
624	Transmembrane domain-containing protein [Brazilian cedratvirus IHUMI]	29.203	219.07	29,46417109	10	57.8
232	Hypothetical protein BRZCDTV_389 [Brazilian cedratvirus IHUMI]	30.749	218.81	28,4124368	7	33.5
310	Ankyrin repeat-containing protein [cedratvirus Ce7-1]	43.116	216.64	25,19925563	12	49.3
253	F-box domain-containing protein [cedratvirus Ce7-1]	22.506	210.95	26,06808408	7	58.1
407	Uncharacterized protein Ce0701_0429 [cedratvirus Ce7-1]	43.645	210.14	26,90394338	22	66
286	Ankyrin repeat-containing protein [Brazilian cedratvirus IHUMI]	44.885	207.97	28,31149673	12	43.2
381	Uncharacterized protein Ce0701_0312 [cedratvirus Ce7-1]	29.905	205.84	25,87207603	12	58.7
440	RNA polymerase II RPB5 subunit [cedratvirus Ce7-1]	26.077	203.64	26,69197591	9	64
355	Hypothetical protein BRZCDTV_303 [Brazilian cedratvirus IHUMI]	26.963	198.78	25,07421239	11	54.8
382	Uncharacterized protein Ce0701_0312 [cedratvirus Ce7-1]	21.629	183.86	24,96992302	10	61
345	Uncharacterized protein Ce0701_0258 [cedratvirus Ce7-1]	14.46	182.27	27,81278928	8	82.7
296	Protein kinase domain-containing protein [cedratvirus Ce7-1]	33.744	181.96	27,53800964	2	70.9
264	RNAse III putative (double-stranded ribonuclease) [cedratvirus Ce7-1]	38.544	178.22	25,97494634	12	46.2
207	Ankyrin repeat-containing protein [cedratvirus Ce7-1]	40.815	177.55	26,07261276	10	50.3
447	F-box domain-containing protein [cedratvirus Ce7-1]	13.865	177.54	27,19426727	3	50.4
557	RNAse III [cedratvirus Ce7-1]	38.024	174.18	26,75503922	26	77.4
210	Hypothetical protein BRZCDTV_378 [Brazilian cedratvirus IHUMI]	42.644	173.98	25,57869339	14	58
695	Mannosyl phosphorylinositol ceramide synthase [cedratvirus Ce7-1]	14.81	171.25	25,00150935	6	52.8
312	Uncharacterized protein Ce0701_0280 [cedratvirus Ce7-1]	40.974	170.24	24,52449481	20	64.1
714	Hypothetical protein BRZCDTV_78 [Brazilian cedratvirus IHUMI]	31.572	167.13	25,29549726	18	63.1
598	Hypothetical protein BRZCDTV_163 [Brazilian cedratvirus IHUMI]	17.434	167.09	28,77182961	9	63.5
802	Uncharacterized protein Ce0701_0108 [cedratvirus Ce7-1]	37.163	165.97	28,23850123	18	58.6
129	Uncharacterized protein Ce0201_0281 [cedratvirus Ce2-1]	30.261	160.45	26,9009107	5	43.9
59	Ankyrin repeat-containing protein [Brazilian cedratvirus IHUMI]	15.08	160.43	26,05465571	6	77.1
327	Beta_helix domain-containing protein [cedratvirus Ce7-1]	56.542	158.75	27,9752725	3	75
176	Hypothetical protein BRZCDTV_403 [Brazilian cedratvirus IHUMI]	25.612	158.39	28,63932228	13	58
594	Hypothetical protein BRZCDTV_163 [Brazilian cedratvirus IHUMI]	17.746	150.15	28,50766881	9	61.6
65	SET domain-containing protein [Brazilian cedratvirus IHUMI]	29.494	149.92	25,87970988	13	61.7
237	Hypothetical protein BRZCDTV_384 [Brazilian cedratvirus IHUMI]	59.32	145.24	30,13671048	17	64.8
417	F-box domain-containing protein [Brazilian cedratvirus IHUMI]	22.042	141.11	28,52620761	5	22.5
285	Uncharacterized protein Ce0701_0431 [cedratvirus Ce7-1]	15.567	135.05	25,78754361	1	15.5
333	DNA-directed RNA Pol II C-term-like phosphatase [cedratvirus Ce7-1]	25.087	130.42	26,96707726	6	58.3
414	TFIIS transcription elongation factor [cedratvirus Ce7-1]	18.955	129.61	26,53813108	10	62.3
810	Uncharacterized protein Ce0201_0219 [cedratvirus Ce2-1]	13.329	129.38	23,81026204	3	47
244	Ankyrin repeat-containing protein [cedratvirus Ce7-1]	43.173	128.47	23,95179558	13	54
157	NTF2-like domain-containing protein [Brazilian cedratvirus IHUMI]	23.923	124.38	31,30645879	12	60.5
527	Uncharacterized protein Ce0701_0191 [cedratvirus Ce7-1]	13.63	123.82	28,49904442	8	82.9
273	Uncharacterized protein Ce0701_0437 [cedratvirus Ce7-1]	12.462	119.91	26,87107086	6	89
242	Ankyrin repeat-containing protein [cedratvirus Ce7-1]	42.767	118.64	25,97226016	11	54
13	Ankyrin repeat-containing protein [Brazilian cedratvirus IHUMI]	12.878	117.37	26,39565659	8	47.7
393	Hypothetical protein BRZCDTV_278 [Brazilian cedratvirus IHUMI]	31.666	114.92	22,81969388	8	35.7
412	Hypothetical protein BRZCDTV_267 [Brazilian cedratvirus IHUMI]	26.784	113.74	26,3551782	11	57.1
799	Hypothetical protein BRZCDTV_18 [Brazilian cedratvirus IHUMI]	9.669	107.32	24,14959208	1	18.9
430	Transmembrane domain-containing protein [Brazilian cedratvirus IHUMI]	17.029	104.14	23,3359019	5	42.6
479	Hypothetical protein BRZCDTV_227 [Brazilian cedratvirus IHUMI]	14.176	102.54	29,49773407	6	31.7
356	Acyl-CoA N-acyltransferase [Brazilian cedratvirus IHUMI]	26.36	100.65	25,96886508	4	42.5
692	WD40 domain-containing protein [cedratvirus Ce7-1]	28.776	99.479	22,86605263	5	20.7
463	Transmembrane domain-containing protein [Brazilian cedratvirus IHUMI]	10.322	98.409	22,71078491	5	55.1
185	Uncharacterized protein Ce0201_0068 [cedratvirus Ce2-1]	27.121	92.622	21,52076149	4	21.5
550	Hypothetical protein BRZCDTV_181 [Brazilian cedratvirus IHUMI]	23.923	92.165	31,86248144	2	33
398	ADPrib_exo_Tox domain-containing protein [cedratvirus Ce2-1]	16.356	91.819	24,87027295	2	27.9
313	Uncharacterized protein Ce0701_0279 [cedratvirus Ce7-1]	28.649	90.883	24,4877739	14	53.8
77	Ankyrin repeat-containing protein [cedratvirus duvanny]	29.309	90.367	20,86135864	5	23.9
781	Methyltransferase [cedratvirus Ce7-1]	36.24	90.157	21,84418805	4	16.2
610	Hypothetical protein BRZCDTV_157 [Brazilian cedratvirus IHUMI]	18.729	89.501	24,2402916	5	47.3
779	Hypothetical protein BRZCDTV_37 [Brazilian cedratvirus IHUMI]	22.162	87.809	24,19650396	5	29.3
357	Acyl-CoA N-acyltransferase [cedratvirus Ce7-1]	28.102	87.483	25,76701291	14	60.3
787	Uncharacterized protein Ce0701_0118 [cedratvirus Ce7-1]	18.544	87.21	28,21001498	7	64
416	F-box domain-containing protein [cedratvirus Ce7-1]	21.82	86.42	27,81792132	15	74.4
788	Uncharacterized protein Ce0701_0117 [cedratvirus Ce7-1]	12.432	84,907	28,89221191	7	59.6
429	Transmembrane domain-containing protein [Brazilian cedratvirus IHUMI]	22.456	84.618	22,08543587	2	11.8
690	Alpha/beta hydrolase [cedratvirus Ce7-1]	34.115	83.334	24,92466863	10	43.8
394	Uncharacterized protein Ce0701_0322 [cedratvirus Ce7-1]	26.309	83.118	23,03069433	4	25.6
83	DNA-directed RNA polymerase subunit RPB2 [Brazilian cedratvirus IHUMI]	136.67	82.202	24,72762553	13	49.1
625	Hypothetical protein BRZCDTV_145 [Brazilian cedratvirus IHUMI]	18.11	81.675	22,88739713	3	24.8
364	Hypothetical protein BQ3484_129 [cedratvirus A11]	99.456	81.437	23,1552302	1	52.9
745	Phosphoglycerate mutase [cedratvirus Ce7-1]	21.179	80.132	22,37655512	1	24.3
271	Transmembrane domain-containing protein [cedratvirus Ce7-1]	36.598	76.555	25,58768972	10	53.4
603	Hypothetical protein BRZCDTV_158 [Brazilian cedratvirus IHUMI]	25.894	75.774	21,68806712	4	23.8
782	Uncharacterized protein Ce0701_0122 [cedratvirus Ce7-1]	71.157	75.352	21,14392726	3	7.8
201	Ubiquitin ligase [Brazilian cedratvirus IHUMI]	15.905	75.323	20,90581449	5	37.2
44	GTP-binding protein [cedratvirus Ce7-1]	13.659	73.639	20,750,199	4	44.2
573	DNA-directed RNA polymerase RPB10 [Brazilian cedratvirus IHUMI]	99.384	72.64	26,60758527	3	63.2
340	DNA-directed RNA Pol II C-term-like phosphatase [cedratvirus Ce7-1]	25.091	72.236	24,99596914	3	50.5
274	Transmembrane domain-containing protein [cedratvirus Ce7-1]	30.199	72.148	23,23042361	5	36.1
112	Uncharacterized protein Ce0701_0410 [cedratvirus Ce7-1]	19.185	71.759	21,61158562	3	13.3
657	5'−3' exoribonuclease [Brazilian cedratvirus IHUMI]	39.065	70.757	24,01133664	3	43.3
838	Uncharacterized protein Ce0701_0091 [cedratvirus Ce7-1]	15.514	70.092	21,94168599	2	23.7
326	Transmembrane domain-containing protein [Brazilian cedratvirus IHUMI]	34.225	69.45	24,39121373	15	59.6
798	Uncharacterized protein Ce0701_0110 [cedratvirus Ce7-1]	11.989	69.342	26,71962865	8	58.3
674	Hypothetical protein BRZCDTV_115 [Brazilian cedratvirus IHUMI]	29.008	67.548	20,87562879	4	20.9
828	DNA topoisomerase IIA [Brazilian cedratvirus IHUMI]	54.397	66.562	20,34713554	4	10.4
318	Uncharacterized protein Ce0701_0276 [cedratvirus Ce7-1]	11.718	66.469	25,65533829	1	27.7
21	F-box domain-containing protein [Brazilian cedratvirus IHUMI]	27.165	65.259	25,79525884	13	50.9
804	Ankyrin repeat-containing protein [cedratvirus Ce7-1]	82.143	65.135	22,30910238	2	18.6
235	Lectin domain-containing protein [cedratvirus Ce7-1]	34.936	63.033	23,1832091	2	17
518	Hypothetical protein ZAZAV_583 [cedratvirus Zaza IHUMI]	18.475	62.937	20,13540936	4	26.3
272	EGF-like domain-containing protein [Brazilian cedratvirus IHUMI]	37.01	62.819	21,51404254	4	15.3
418	RecD/TraA family helicase repair protein [cedratvirus Ce7-1]	76.053	62.613	21,0296491	4	7.2
803	Hypothetical protein BRZCDTV_14 [Brazilian cedratvirus IHUMI]	32.944	61.97	25,38799604	10	41.7
68	Translation elongation factor [cedratvirus A11]	46.14	61.297	21,47631836	4	9.7
127	Hypothetical protein BRZCDTV_433 [Brazilian cedratvirus IHUMI]	28.741	60.441	24,44642194	3	35.7
497	Hydrolase-like domain-containing protein [cedratvirus Ce7-1]	22.002	56.843	21,48840332	4	25.5
380	Hypothetical protein BRZCDTV_290 [Brazilian cedratvirus IHUMI]	10.231	56.476	23,51665878	6	64.4
660	Uncharacterized protein Ce0701_0148 [cedratvirus Ce7-1]	32.736	54.854	25,98714892	10	42.2
372	Transmembrane domain-containing protein [cedratvirus Ce7-1]	17.875	53.991	28,22165108	2	28.2
448	RNA polymerase II RPB5 subunit [cedratvirus Ce7-1]	26.183	53.536	26,33921814	7	54.4
211	Ankyrin repeat-containing protein [cedratvirus Ce7-1]	42.027	53.513	31,84666667	11	52.5
365	Divergent major capsid protein [cedratvirus lena]	54.217	52.685	21,47901535	3	7.7
317	Hypothetical protein BRZCDTV_326 [Brazilian cedratvirus IHUMI]	43.549	52.656	25,33771833	15	41
167	AP-endonuclease [Brazilian cedratvirus IHUMI]	50.515	51.646	20,08758036	4	10.3
724	Hypothetical protein BRZCDTV_68 [Brazilian cedratvirus IHUMI]	13.832	50.42	23,81411743	8	69.2
302	Ribonuclease H-like protein [Brazilian cedratvirus IHUMI]	20.247	48.401	21,0942564	4	27.1
472	Glycosyltransferase family 2 [Brazilian cedratvirus IHUMI]	47.334	46.757	20,24222438	3	6.9
404	Hypothetical protein BRZCDTV_272 [Brazilian cedratvirus IHUMI]	16.765	46.496	20,98172283	2	21.4
361	Hypothetical protein BRZCDTV_300 [Brazilian cedratvirus IHUMI]	24.16	45.893	24,88035266	7	50.2
786	PD-(D/E)XK nuclease [cedratvirus Ce7-1]	28.187	45.459	20,65314102	3	13.7
219	F-box domain-containing protein [cedratvirus Ce7-1]	23.775	45.174	20,67233658	4	23.2
601	Transmembrane domain-containing protein [Brazilian cedratvirus IHUMI]	12.499	44.016	26,43851852	3	48.3
373	Hypothetical protein BRZCDTV_297 [Brazilian cedratvirus IHUMI]	72.442	43.954	24,78686841	1	74.2
626	Hypothetical protein Cduv_22 [cedratvirus duvanny]	12.351	43.916	25,79482714	6	47.7
469	Hypothetical protein BRZCDTV_235 [Brazilian cedratvirus IHUMI]	25.515	43.783	20,82987849	3	16.9
442	Hypothetical protein BRZCDTV_246 [Brazilian cedratvirus IHUMI]	50.79	42.721	22,43225733	1	37.9
693	Uncharacterized protein Ce0701_0376 [cedratvirus Ce7-1]	94.825	42.415	24,14972178	2	32.9
360	Acyl-CoA N-acyltransferase [Brazilian cedratvirus IHUMI]	26.287	42.384	22,87440681	4	42.5
615	ATP-dependent DNA ligase [Brazilian cedratvirus IHUMI]	46.883	42.342	23,90722783	12	26.7
238	RRM domain-containing protein [cedratvirus Ce7-1]	25.76	42.282	20,80888875	2	22.2
368	Transmembrane domain-containing protein [cedratvirus Ce7-1]	13.22	41.707	23,66727638	2	37.8
383	Uncharacterized protein Ce0701_0313 [cedratvirus Ce7-1]	31.976	39.561	24,50901413	14	59.4
362	Transmembrane domain-containing protein [cedratvirus Ce7-1]	72.554	38.761	27,86993027	1	12.5
507	Uncharacterized protein Ce0701_0181 [cedratvirus Ce7-1]	18.801	38.409	27,78802427	8	49.7
209	Hypothetical protein BRZCDTV_379 [Brazilian cedratvirus IHUMI]	43.485	38.189	23,75448926	8	40.9
672	5'−3' exoribonuclease [Brazilian cedratvirus IHUMI]	26.132	38.116	28,54252243	10	33.8
193	Hypothetical protein BRZCDTV_394 [Brazilian cedratvirus IHUMI]	31.923	37.478	27,30957921	6	84.1
199	Ankyrin repeat-containing protein [cedratvirus Ce7-1]	20.407	37.379	21,58468564	2	12.4
293	DEAD/SNF2 DNA/RNA helicase [Brazilian cedratvirus IHUMI]	34.224	37.147	24,42343521	13	46.1
307	Protein kinase domain-containing protein [cedratvirus Ce7-1]	33.744	36.985	23,85439746	2	70.9
515	Serine/Threonine protein kinase [cedratvirus Ce7-1]	62.353	36.694	22,57287916	12	23.5
276	Transmembrane domain-containing protein [cedratvirus Ce7-1]	23.245	36.604	22,89136124	3	29.2
747	Uncharacterized protein Ce0201_0009 [cedratvirus Ce2-1]	16.237	34.638	26,8409818	6	36
390	Hypothetical protein BRZCDTV_281 [Brazilian cedratvirus IHUMI]	15.077	32.926	20,66976452	2	20.6
84	Endoribonuclease L-PSP/chorismate mutase-like protein [Brazilian cedratvirus IHUMI]	19.649	32.908	26,71621831	2	44.7
294	DEAD/SNF2 DNA/RNA helicase [cedratvirus Ce7-1]	15.146	32.573	23,40559959	2	69.6
720	Uncharacterized protein Ce0701_0333 [cedratvirus Ce7-1]	19.575	32.133	23,0743262	6	40.7
800	Hypothetical protein BRZCDTV_17 [Brazilian cedratvirus IHUMI]	84.726	31.955	24,19575055	3	77.2
353	Helicase nuclease [cedratvirus Ce7-1]	22.229	31.951	26,502,189	7	51.9
304	Uncharacterized protein Ce0701_0276 [cedratvirus Ce7-1]	11.467	31.702	25,24331474	1	20
323	Hypothetical protein BRZCDTV_321 [Brazilian cedratvirus IHUMI]	23.45	30.945	29,03521156	3	16.7
116	Hypothetical protein BRZCDTV_442 [Brazilian cedratvirus IHUMI]	16.342	30.704	20,40,846697	2	15.3
792	Uncharacterized protein Ce0701_0114 [cedratvirus Ce7-1]	18.605	28.684	20,4864502	2	12.7
806	Deoxynucleoside monophosphate kinase [cedratvirus Ce7-1]	24.151	28.052	23,21419716	9	44.7
574	Caspase-like protein [Brazilian cedratvirus IHUMI]	28.759	27.478	24,33054161	4	44.6
311	Ankyrin repeat-containing protein [cedratvirus Ce7-1]	42.502	27.302	24,14866702	9	33
519	Hypothetical protein Cbor_555 [cedratvirus borely]	94.982	27.282	20,94904709	1	12.9
439	F-box domain-containing protein [Brazilian cedratvirus IHUMI]	13.079	26.895	25,70086225	4	41.4
12	Ribonuclease H [Brazilian cedratvirus IHUMI]	18.06	26.379	20,4560585	2	14.3
675	Uncharacterized protein Ce0701_0164 [cedratvirus Ce7-1]	26.381	25.981	20,10263157	2	12.8
389	Transmembrane domain-containing protein [Brazilian cedratvirus IHUMI]	17.166	25.26	23,7800808	10	50
388	Uncharacterized protein Ce0701_0318 [Cedratvirus Ce7-1]	15.703	24.959	23,95928256	7	60.9
100	Transmembrane domain-containing protein [Brazilian cedratvirus IHUMI]	20.128	24.95	25,17103831	5	21.6
359	Acyl-CoA N-acyltransferase [Brazilian cedratvirus IHUMI]	25.96	24.732	23,87978617	9	39.2
278	Uncharacterized protein Ce0701_0433 [cedratvirus Ce7-1]	17.313	24.707	20,99335384	2	13.2
42	Hypothetical protein BRZCDTV_498 [Brazilian cedratvirus IHUMI]	86.448	24.669	21,33561643	2	25
45	Uncharacterized protein Ce0701_0382 [cedratvirus Ce7-1]	14.441	24.341	21,08392715	2	12.9
558	Hypothetical protein BQ3484_556 [cedratvirus A11]	13.707	23.624	21,03420575	1	12.2
552	Hypothetical protein BRZCDTV_180 [Brazilian cedratvirus IHUMI]	48.719	23.502	25,73977661	6	44.5
516	Hypothetical protein Cbor_435 [cedratvirus borely]	49.965	23.145	23,31884511	12	31.2
646	Uncharacterized protein Ce0701_0148 [cedratvirus Ce7-1]	32.881	21.738	25,12048531	7	40.8
841	Hypothetical protein BRZCDTV_2 [Brazilian cedratvirus IHUMI]	13.074	21.032	24,85709953	6	56.9
386	Hypothetical protein BRZCDTV_285 [Brazilian cedratvirus IHUMI]	22.074	20.586	21,79217211	10	58
762	Alpha/beta hydrolase [cedratvirus borely]	27.427	20.278	19,56126785	1	24.6
801	Transmembrane domain-containing protein [Brazilian cedratvirus IHUMI]	15.818	20.179	24,23126666	8	42.2
700	Lipocalin/cytosolic fatty-acid binding domain-containing protein [cedratvirus Ce7-1]	18.121	19.824	27,90670458	1	63.8
670	Cyclin dependent kinase [cedratvirus Ce7-1]	41.715	18.768	22,2694308	2	11.6
375	Divergent Major Capsid Protein [cedratvirus Ce7-1]	53.984	18.642	24,13393211	10	30.3
387	Patatin-like phospholipase [cedratvirus Ce7-1]	30.064	17.495	22,1608874	6	26.6
37	Hypothetical protein BRZCDTV_503 [Brazilian cedratvirus IHUMI]	91.397	16.936	23,85832723	4	56.5
35	Hypothetical protein Ce0701_0388 [cedratvirus Ce7-1]	90.578	16.677	22,11165301	1	43.2
468	Transmembrane domain-containing protein [Brazilian cedratvirus IHUMI]	21.418	16.066	21,76283582	2	20
405	F-box domain-containing protein [Brazilian cedratvirus IHUMI]	18.611	15.657	23,21444575	6	53.8
584	Caspase-like protein [Brazilian cedratvirus IHUMI]	28.745	15.565	22,87681071	3	36.3
539	TFIIB transcription initiation factor [cedratvirus Ce7-1]	21.079	15.515	23,34710248	1	23.7
766	Hypothetical protein BRZCDTV_44 [Brazilian cedratvirus IHUMI]	14.057	14.655	24,76990954	4	47.6
756	Alpha/beta hydrolase [Brazilian cedratvirus IHUMI]	30.275	14.565	22,50302696	4	35
528	Protein containing kinase domain [Brazilian cedratvirus IHUMI]	34.999	14.354	21,88711929	7	30.5
204	Hypothetical protein BRZCDTV_383 [Brazilian cedratvirus IHUMI]	25.554	14.292	22,24746513	6	51.6
824	Ankyrin repeat-containing protein [cedratvirus Ce7-1]	19.785	13.893	21,58187358	2	16.1
224	F-box domain-containing protein [cedratvirus Ce7-1]	23.568	13.462	23,2784551	5	24.3
197	Hypothetical protein BRZCDTV_389 [Brazilian cedratvirus IHUMI]	65.323	13.452	27,11423937	4	62.5
723	Beta-1,3-N-acetylglucosaminyltransferase lunatic fringe-like [Brazilian cedratvirus IHUMI]	39.962	13.413	23,38363266	4	11.4
451	Uncharacterized protein Ce0701_0228 [cedratvirus Ce7-1]	16.856	13.042	29,03384336	4	23.9
541	Uncharacterized protein Ce0701_0203 [cedratvirus Ce7-1]	24.039	12.872	28,55572255	2	32.9
280	Uncharacterized protein Ce0701_0437 [cedratvirus Ce7-1]	12.448	12.106	23,07018026	4	82.6
92	Ankyrin repeat-containing protein [Brazilian cedratvirus IHUMI]	15.108	11.379	22,81090864	4	54.2
454	Transmembrane domain-containing protein [cedratvirus Ce7-1]	18.266	11.037	22,38300387	2	16
58	Hypothetical protein BRZCDTV_486 [Brazilian cedratvirus IHUMI]	15.013	10.781	21,90730794	3	31.3
654	PolyA polymerase reg subunit [cedratvirus Ce7-1]	26.708	10.589	21,39780299	5	24.2
371	Hypothetical protein BRZCDTV_290 [Brazilian cedratvirus IHUMI]	91.446	10.514	23,78207397	5	77.2
434	F-box domain-containing protein [Brazilian cedratvirus IHUMI]	25.204	10.324	22,13642438	5	31.1
358	Hypothetical protein BRZCDTV_300 [Brazilian cedratvirus IHUMI]	28.011	10.174	22,83262189	3	29.9
464	Hypothetical protein BRZCDTV_235 [Brazilian cedratvirus IHUMI]	22.828	5.865	20,78565407	3	19.4
305	DEAD/SNF2 DNA/RNA helicase [cedratvirus Ce7-1]	21.879	3.708	22,59363238	2	42.9
708	Hypothetical protein BRZCDTV_84 [Brazilian cedratvirus IHUMI]	17.511	3.072	20,84053421	2	15.6

A significant proportion of these proteins (30%) were classified as hypothetical or uncharacterized, including proteins with transmembrane domains (Fig. 1a; see Table S1 at https://www.giantviruses.com/sup-material-of-papers/sup-material-the-proteomics-of-the-giant-cedratvirus-particles-reveals-unique-and-shared-features-with-pitho-like-viruses). The identified proteins also participate in various metabolic pathways, such as lipid, nucleotide, and carbohydrate metabolism, suggesting potential roles in manipulating the host environment to benefit viral propagation. Notably, two major capsid protein (MCP) genes were detected, and both corresponding proteins were identified through proteomic analysis, highlighting their essential roles in virion assembly and structural architecture. Furthermore, 172 proteins associated with the host *Acanthamoeba castellanii* were identified, indicating that host proteins may be incorporated in the viral particles during the late step of the cycle (Table 2). We acknowledge that it is possible that some of the detected proteins may result from residual contamination or incidental association with the virion. However, we performed two rounds of sucrose cushion purification to minimize such contaminants, and we carefully interpret our proteomic data with this consideration in mind.

**TABLE 2:** *Acanthamoeba* proteins identified in association with cedratvirus pambiensis purified particles after valid value filter and excluding reverse sequences and “only identified by site” entries

Protein IDs	Protein description	Mol. weight (kDa)	Sequence coverage (%)	Unique peptides	LFQ_1	LFQ_2	LFQ_3
gi|470523558|ref|XP_004355037.1|	DIL domain-containing protein	77.039	2.5	1	30,24059	NaN	30,13038
gi|470410604|ref|XP_004336285.1|	Eukaryotic porin protein	33.585	76.5	20	27,36986	27,50718	27,40617
gi|470388901|ref| XP_004334426.1|	Uncharacterized protein ACA1_023990, partial	18.53	47.2	7	26,42153	26,73918	26,54712
gi|470512969|ref|XP_004352564.1|	Actin-1, putative	41.675	65.9	19	26,26202	26,36765	26,56677
gi|302393707|sp|P49633.2|RL40_ACACA	Ubiquitin-ribosomal protein eL40 fusion protein	14.766	33.6	2	26,47314	25,93188	26,39098
gi|470437283|ref|XP_004338972.1|	Adenine nucleotide translocator, putative	34.021	39.3	15	25,87554	26,06163	25,93984
gi|470426286|ref|XP_004337881.1|	Chorismate mutase subfamily protein	20.759	34	4	24,86486	24,71639	25,20083
gi|470527464|ref|XP_004357162.1|	DEAD/DEAH box helicase domain-containing protein, partial	64.87	60.6	34	24,90049	24,93531	24,89458
gi|470482936|ref|XP_004343659.1|	Uncharacterized protein ACA1_099260	44.325	53.5	22	24,75573	24,77047	24,76754
gi|470460317|ref|XP_004341448.1|	Core histone h2a/h2b/h3/h4 superfamily protein	17.322	9.8	2	24,63616	24,93233	24,48401
gi|470408994|ref|XP_004336147.1|	Retrovirus-related Pol polyprotein from transposon TNT 1-94, putative	50.373	2	1	24,65048	24,92452	24,22266
gi|909719706|gb|AKT93988.1|	H(+)-transporting ATPase subunit 9 (mitochondrion)	82.459	12.7	1	24,72931	24,33668	24,5657
gi|470373083|ref|XP_004332988.1|	Peroxidase	36.915	28.3	3	24,40178	24,6019	24,3201
gi|470403734|ref|XP_004335740.1|	Uncharacterized protein ACA1_305680, partial	17,346	7,9	1	24,24663	24,7681	24,29551
gi|470418522|ref|XP_004337098.1|	Cyclophilin, putative	18.1	43.9	7	24,26019	24,55274	24,35763
gi|56405382|sp|Q95VF7.3|PRO1B_ACACA	Acidic profilin IB	12.938	27.8	3	24,45883	24,30536	24,35043
gi|470409867|ref|XP_004336226.1|	Histone H2A variant, putative	14.104	22.9	3	23,88976	23,96517	23,86938
gi|470388239|ref|XP_004334366.1|	C8 sterol isomerase	21.546	21.5	4	23,51629	24,41209	23,78227
gi|470516619|ref|XP_004353105.1|	C2 domain-containing protein	14.171	49.6	5	23,56209	24,12715	23,91752
gi|470484671|ref|XP_004344091.1|	Eukaryotic initiation factor 4A, putative	46.377	57.5	20	23,65086	23,63223	23,68667
gi|470433657|ref|XP_004338597.1|	Peptidase M16 family protein	53.866	48.4	15	23,54215	23,53089	23,61796
gi|470393666|ref|XP_004334908.1|	Cytochrome c oxidase	17.896	26.1	4	23,32924	23,76274	23,3692
gi|6647494|sp|Q37370.1|COX1_ACACA	Cytochrome c oxidase subunit 1+2	99.213	7.8	6	23,42522	23,27938	23,69221
gi|470515801|ref|XP_004352977.1|	Sar1 family small GTPase	21.68	34.2	5	23,31631	23,52325	23,46588
gi|470447037|ref|XP_004340024.1|	Prohibitin 2, putative	32.271	28.9	8	23,44228	23,52468	23,31271
gi|470456089|ref|XP_004341029.1|	Fascin subfamily protein	14.443	41.7	7	23,10238	23,43059	23,45312
gi|470529620|ref|XP_004368102.1|	Prohibitin PHB1, putative	31.311	40.6	9	23,27327	23,39937	23,28673
gi|470401395|ref|XP_004335588.1|	Uncharacterized protein ACA1_359140	65.257	2.2	1	24,05009	22,28452	23,34652
gi|470466481|ref|XP_004341712.1|	Citrate transport family protein	34.287	14.8	4	23,24195	23,14513	23,20725
gi|470426988|ref|XP_004337951.1|	Uncharacterized protein ACA1_297990	15.949	6.1	1	22,5991	23,30966	23,32113
gi|470531418|ref|XP_004358065.1|	Uncharacterized protein ACA1_074190	18.951	16.3	1	23,05793	22,74651	23,37066
gi|470530880|ref|XP_004368325.1|	Peptidase M16 inactive domain-containing protein	63.091	23.2	14	22,92133	23,24747	22,96856
gi|1878344716|gb|QLM05583.1|	Inosineuridine preferring nucleoside hydrolase	40.867	16.8	5	22,93404	23,16197	22,95635
gi|470381459|ref|XP_004333780.1|	EF hand domain-containing protein	24.933	23.3	3	22,94367	22,8694	23,21542
gi|470395572|ref|XP_004335087.1|	Peroxidase	134.07	27.3	26	22,98017	23,05475	22,9362
gi|470426871|ref|XP_004337942.1|	Eukaryotic initiation factor 5a, putative	16.695	19.9	2	22,77211	23,16558	22,92074
gi|470409926|ref|XP_004336232.1|	GTP-binding protein	22.059	5.6	1	22,6285	23,01968	22,81402
gi|470475956|ref|XP_004342208.1|	LIM domain-containing protein	15.264	14.1	1	22,55459	23,10252	22,72228
gi|470510518|ref|XP_004351540.1|	MAPEG domain-containing protein	15.178	18.3	3	22,80069	22,98919	22,56858
gi|470443766|ref|XP_004339681.1|	RNA-binding protein	57.752	20.7	8	22,48375	22,99397	22,78969
gi|470444060|ref|XP_004339708.1|	Rho family, small GTP-binding protein Rac3, putative	21.686	30.1	4	22,60779	22,7918	22,72586
gi|470375092|ref|XP_004333159.1|	High molecular weight heat shock protein	72.287	42.2	20	22,64181	22,85363	22,61131
gi|470414438|ref|XP_004336688.1|	LIM domain-containing protein	18.989	23.7	3	22,24016	23,23182	22,61778
gi|470469092|ref|XP_004341966.1|	Aldehyde dehydrogenase (NAD) family superfamily protein	55.195	18.7	7	22,73705	22,65851	22,66338
gi|470509236|ref|XP_004349728.1|	Rho family, small GTP-binding protein Rac3, putative	21.43	31.6	3	22,78137	22,54948	22,63252
gi|33358312|gb|AAQ16627.1|	Ubiquitin-like protein Ublp94.4	94.409	2.6	1	22,56614	22,73159	22,45861
gi|470467738|ref|XP_004341828.1|	Elongation factor 1-alpha, putative	41.797	14.9	2	22,54536	22,28949	22,90176
gi|470422038|ref|XP_004337460.1|	Peptidylprolyl cis-trans isomerase	18.521	25.6	4	22,553	22,59099	22,56312
gi|470520651|ref|XP_004353625.1|	Malate dehydrogenase	45.09	44.7	14	22,64926	22,64777	22,40367
gi|470454205|ref|XP_004340817.1|	Uncharacterized protein ACA1_040810	86.587	22.5	2	22,51458	22,58422	22,52834
gi|470373923|ref|XP_004333065.1|	Copper/zinc superoxide dismutase	16.319	55.4	6	22,44995	22,63301	22,41191
gi|470525570|ref|XP_004356622.1|	Mitochondrial phosphate transporter, putative	34.929	22.8	6	22,66303	22,16023	22,66627
gi|470448072|ref|XP_004340125.1|	Leucine rich repeat domain-containing protein	103.73	1.7	2	22,50383	22,38753	22,55852
gi|855099064|gb|AKN79947.1|	Sorting assembly machinery 50 kDa subunit	48.127	21.5	7	22,63969	22,46019	22,30747
gi|470381584|ref|XP_004333791.1|	Gar1 protein RNA-binding region protein	21.336	17.2	3	22,45643	22,51241	22,41158
gi|157833559|pdb|1PRQ|A	Chain A, profilin IA	12.952	20	1	22,44582	22,34557	22,51292
gi|470384632|ref|XP_004334068.1|	Uncharacterized protein ACA1_025730, partial	21.563	32.1	8	22,35714	22,50543	22,43578
gi|470413895|ref|XP_004336630.1|	Syntaxin 6, putative	15.932	11.8	2	22,45588	22,14143	22,69994
gi|470427997|ref|XP_004338048.1|	Peptidase S8 and S53 subtilisin kexin sedolisin, putative	44.359	35	7	22,6054	22,26178	22,2932
gi|470521501|ref|XP_004353726.1|	LIM domain-containing protein	13.774	27.2	2	22,30042	22,47914	22,35512
gi|470458139|ref|XP_004341211.1|	Filamin repeat domain-containing protein	88.769	15.1	10	22,32811	22,34573	22,43311
gi|470492041|ref|XP_004345964.1|	Peptidylprolyl isomerase FKBP12, putative	11.774	33.9	3	NaN	22,3555	22,31797
gi|470429295|ref|XP_004338185.1|	Hypothetical protein ACA1_178210	26.525	36.1	5	22,49782	22,14855	22,31163
gi|470380223|ref|XP_004333668.1|	Ran, putative	24.106	22.5	4	21,89685	22,99229	22,06533
gi|562063|gb|AAD11851.1|	ORF25 (mitochondrion)	14.388	20.2	2	22,17026	NaN	22,396
gi|470423031|ref|XP_004337554.1|	P18, putative	15.161	30.6	5	NaN	22,66173	21,89208
gi|385281908|gb|AFI57874.1|	Beta-tubulin, partial	35.204	9.1	3	NaN	21,85365	22,6846
gi|909719709|gb|AKT93991.1|	H(+)-transporting ATPase subunit 1 (mitochondrion)	57.645	19.2	10	22,18138	22,27247	22,34302
gi|470508831|ref|XP_004349636.1|	Pyruvate dehydrogenase complex dihydrolipoamide acetyltransferase	53.011	8.2	4	22,30516	22,1887	22,29737
gi|470518821|ref|XP_004353386.1|	Uncharacterized protein ACA1_123770	13.5	15.2	1	22,12569	22,15734	22,50082
gi|470430846|ref|XP_004338340.1|	Uncharacterized protein ACA1_203710	22.007	28.7	5	22,27915	22,23216	22,12559
gi|470519565|ref|XP_004353478.1|	rRNA pseudouridine synthase, putative	58.498	10.1	4	22,30941	22,11728	22,20661
gi|470409794|ref|XP_004336219.1|	Protein kinase domain-containing protein	77.397	2	2	22,28102	NaN	22,10623
gi|470375951|ref|XP_004333241.1|	RNA recognition motif domain-containing protein	54.021	24.9	10	21,94628	22,24496	22,26347
gi|470446257|ref|XP_004339937.1|	Exported protein, putative	21.838	15.4	2	22,30791	21,998	22,14622
gi|470377432|ref|XP_004333384.1|	LSM domain-containing protein	14.673	14	2	22,61255	22,17643	21,66049
gi|470530751|ref|XP_004368305.1|	Gamma carbonic anhydrase	29.786	8.1	2	22,09088	22,16809	22,15462
gi|470508654|ref|XP_004349608.1|	Zn-finger in Ran-binding protein and other domain-containing protein	20.926	36.2	4	21,85034	22,43428	22,03564
gi|470449957|ref|XP_004340336.1|	Uncharacterized protein ACA1_371630	24.86	22.4	4	21,86389	22,32056	22,09136
gi|470491417|ref|XP_004345890.1|	PXMP2/4 family protein 3, putative	21.198	18.8	2	22,30316	21,68355	22,25825
gi|470509447|ref|XP_004367727.1|	Autophagy-related protein 27 protein	28.843	37.3	10	21,91984	22,33764	21,88755
gi|440802468|gb|ELR23397.1|	Chorismate mutase, putative	20.869	28.2	4	21,8933	22,0676	22,08352
gi|470506064|ref|XP_004348701.1|	Ras family protein	24.602	21.8	5	21,94806	21,79074	22,23362
gi|470519921|ref|XP_004353526.1|	FGGAP repeat domain-containing protein	48.075	14.9	6	21,96349	21,99824	22,00446
gi|470380103|ref|XP_004333657.1|	Gamma CA3 (gamma carbonic anhydrase 3), putative	30.787	28.4	7	22,07715	22,13389	21,71272
gi|470510220|ref|XP_004351498.1|	I/LWEQ domain-containing protein	70.416	6.5	4	21,94521	21,95326	21,80982
gi|470393565|ref|XP_004334896.1|	Ribosomal protein L6, putative	21.447	22.2	5	21,89149	21,98694	21,81511
gi|881635|gb|AAA93068.1|	Arp3	48.635	19	8	21,8459	22,21715	21,51956
gi|470521487|ref|XP_004353724.1|	Histone H4, putative	12.062	18.2	2	21,75788	22,18435	21,61612
gi|440803274|gb|ELR24182.1|	Cell division control protein 2b, putative	34.498	7.6	2	NaN	21,5582	22,05904
gi|470384600|ref|XP_004334064.1|	Fibrillarin, putative	32.559	21.8	5	21,87763	21,85582	21,68821
gi|470377562|ref|XP_004333398.1|	C2 domain-containing protein	70.216	10.1	6	21,47412	21,98999	21,92644
gi|470521351|ref|XP_004353703.1|	RabE family small GTPase, partial	17.38	21.3	3	21,83881	21,54	21,89586
gi|470379314|ref|XP_004333569.1|	Uncharacterized protein ACA1_257560	16.537	13.4	2	21,65652	21,84357	21,7615
gi|470413688|ref|XP_004336605.1|	Atp-dependent rna helicase ddx6, putative	46.821	23.8	7	21,74481	21,66917	21,75499
gi|470394048|ref|XP_004334951.1|	Small G-protein	20.63	6	1	21,88372	21,58651	21,46079
gi|470493083|ref|XP_004346472.1|	Cholinesterase	55.495	2.6	1	21,68564	21,44319	21,75316
gi|470449988|ref|XP_004340340.1|	BAR domain-containing protein	46.091	27.1	7	21,66887	21,30697	21,58284
gi|470444553|ref|XP_004339766.1|	Ribosomal protein S8, putative	14.776	20.8	2	21,46154	21,63139	21,45899
gi|470419216|ref|XP_004337172.1|	Mitochondrial import receptor subunit tom40, putative	39.771	11.4	4	21,69098	21,36485	21,48301
gi|470389887|ref|XP_004334520.1|	CMF receptor CMFR1, putative	51.865	20.6	5	21,45332	21,42429	21,62078
gi|470424802|ref|XP_004337738.1|	Casein kinase II subunit alpha, putative	40.052	5.8	2	NaN	21,55112	21,44374
gi|470446444|ref|XP_004339960.1|	SAC3/GANP family protein, partial	72.254	3.4	2	NaN	21,55651	21,43252
gi|470373091|ref|XP_004332989.1|	Peroxidase	23.233	18.1	1	21,46837	21,91903	21,08459
gi|82470775|gb|AAL87229.3|AF480890_1	Metacaspase	50.248	16.9	5	21,41803	21,99948	21,02384
gi|470524449|ref|XP_004356482.1|	Protein phosphatase 2A regulatory B subunit	48.732	9	3	21,39535	21,53365	21,49984
gi|470466515|ref|XP_004341716.1|	Uncharacterized protein ACA1_198450	85.486	15.3	9	21,093	21,48984	21,84142
gi|2169164908|pdb|7RTX|A	Chain A, actophorin	15.422	10.2	1	21,51494	21,0176	21,85829
gi|470420396|ref|XP_004337289.1|	Eukaryotic translation elongation factor 2, putative	93.341	14.8	9	21,25621	21,35875	21,72538
gi|470455700|ref|XP_004340986.1|	Vacuolar proton pump d subunit, putative	35.204	12.2	3	21,29793	21,42019	21,61149
gi|470391452|ref|XP_004334670.1|	ATP:L-methionine S-adenosyltransferase	42.531	12.4	4	NaN	21,63485	21,20467
gi|470526148|ref|XP_004356708.1|	Peroxin 7 (Pex7), putative	36.454	4	1	NaN	21,25442	21,55126
gi|470455432|ref|XP_004340951.1|	Ubiquinol-cytochrome c reductase complex 14 kDa protein	14.346	21.3	2	21,37809	21,67124	21,13959
gi|470415165|ref|XP_004336758.1|	Uncharacterized protein ACA1_390820	33.151	14.6	4	21,37465	21,49116	21,29261
gi|470527031|ref|XP_004367812.1|	1-aminocyclopropane-1-carboxylate deaminase	51.225	4.3	1	21,24928	21,63338	21,25315
gi|470450829|ref|XP_004340430.1|	Elongation factor one alpha, somatic form, putative, partial	35.895	12.8	1	21,26184	21,48104	21,31904
gi|470475309|ref|XP_004342146.1|	ABC2 type transporter superfamily protein	78.702	4.4	3	NaN	21,48055	21,20174
gi|470509344|ref|XP_004350025.1|	Carrier superfamily protein	37.082	14.2	4	21,36068	21,17085	21,43695
gi|470468808|ref|XP_004341938.1|	Domain found in disheveled, egl10, and pleckstrin domain-containing protein	41.043	19.1	6	21,42977	21,23894	21,27407
gi|470423572|ref|XP_004337611.1|	Ras-related protein Rab-2A, putative	22.613	18	3	21,12244	21,22635	21,56118
gi|470444765|ref|XP_004339792.1|	ARP2/3 complex 34 kDa subunit, putative	33.355	21.8	6	20,50368	21,39336	22,01277
gi|470553140|ref|XP_004367444.1|	Alkaline phosphatase family subfamily protein	62.305	2.6	1	20,85673	21,69943	21,30825
gi|470525692|ref|XP_004356641.1|	Protein phosphatase 1, catalytic subunit, alpha, putative	41.035	23.5	6	20,70852	21,66132	21,25828
gi|470401895|ref|XP_004335633.1|	Uncharacterized protein ACA1_037930	21.013	11.3	1	20,77193	21,11579	21,73826
gi|470382483|ref|XP_004333878.1|	Uncharacterized protein ACA1_273770	39.011	25.9	7	20,51384	22,21134	20,80861
gi|470519360|ref|XP_004353460.1|	Uncharacterized protein ACA1_075000	10.377	22	2	21,11858	21,28557	21,07333
gi|470388596|ref|XP_004334397.1|	Protein translation factor, putative	12.289	17.3	2	21,16202	21,29283	20,94714
gi|470510488|ref|XP_004351535.1|	Actin-related protein ARPC3, putative	18.06	14.4	2	21,22199	20,80183	21,3531
gi|470398314|ref|XP_004335307.1|	Sterol 24C-methyltransferase	39.022	15.3	3	21,13295	21,1091	21,11343
gi|470446485|ref|XP_004339965.1|	NADH dehydrogenase, putative	12.6	11.7	1	NaN	21,29227	20,93027
gi|470396670|ref|XP_004335168.1|	BAT1 protein	51.693	14.2	5	20,9047	21,37528	20,99404
gi|470515115|ref|XP_004352880.1|	Uncharacterized protein ACA1_069360	18.977	22.4	3	20,92731	20,73265	21,37311
gi|470421137|ref|XP_004337362.1|	Sec1 family protein, partial	50.699	5.3	2	NaN	21,14283	20,86914
gi|470373753|ref|XP_004333044.1|	Ribosomal protein S5, putative	27.129	25.9	5	20,1706	21,43405	21,34933
gi|470526296|ref|XP_004356732.1|	Vacuolar proton ATPase, putative	92.622	7.4	5	20,90205	20,98197	21,01025
gi|470449103|ref|XP_004340234.1|	Tetratricopeptide repeat domain-containing protein	39.975	5.3	2	20,84276	20,6591	21,37571
gi|470448359|ref|XP_004340152.1|	Carnitine/acylcarnitine translocase, putative	28.804	14.4	3	21,07261	20,7376	20,97967
gi|470455770|ref|XP_004340994.1|	Zn-finger in Ran-binding protein and other domain-containing protein	25.801	21	2	NaN	21,2454	20,58077
gi|470428729|ref|XP_004338121.1|	Uncharacterized protein ACA1_025540	44.626	9.1	3	NaN	20,71129	21,00319
gi|470425113|ref|XP_004337775.1|	Uncharacterized protein ACA1_379440	11.043	26.3	2	20,98537	21,1354	20,39665
gi|470399342|ref|XP_004335405.1|	Ras subfamily protein	28.833	16.3	2	21,00265	20,4997	20,96148
gi|470467441|ref|XP_004341795.1|	Guanine nucleotide-binding protein beta subunit, putative	35.215	11.8	3	21,20437	20,73397	20,50038
gi|470465131|ref|XP_004341583.1|	Ribosomal protein S9, putative	16.561	8	1	20,59295	20,78596	21,00086
gi|470407043|ref|XP_004336018.1|	Vacuolar protein sorting-associated protein 45, putative	63.206	6.8	4	20,76085	20,53032	20,8753
gi|470413626|ref|XP_004336599.1|	Uncharacterized protein ACA1_321030	56.146	14.2	5	20,39509	21,20305	20,49999
gi|470523597|ref|XP_004355043.1|	Ras-related protein Rab-7, putative	20.543	12.4	2	20,78101	20,56932	20,73942
gi|470427289|ref|XP_004337981.1|	Catalase, putative	55.346	3.5	1	20,77411	20,8285	20,46862
gi|470530269|ref|XP_004368214.1|	Small nuclear ribonucleoprotein Sm D1, putative	13.41	10.4	1	20,85148	20,76709	20,351
gi|470389022|ref|XP_004334436.1|	LIM domain-containing protein	39.651	8.5	2	20,50116	20,31296	21,07679
gi|470484138|ref|XP_004344039.1|	Peroxin 5 (Pex5), putative	87.461	4.5	3	20,54633	20,71087	20,59067
gi|470443710|ref|XP_004339676.1|	Elongation factor SIII p15 subunit, putative	11.513	38.8	4	20,29345	20,75654	20,50814
gi|470532050|ref|XP_004358287.1|	Eukaryotic ribosomal protein L18, putative	20.902	12.9	2	20,70455	20,00031	20,79257
gi|470446370|ref|XP_004339951.1|	Uncharacterized protein ACA1_208120	36.026	4.3	1	20,58536	19,99507	20,89467
gi|470468369|ref|XP_004341892.1|	DNA repair protein RAD51, putative	37.261	21.6	5	20,26819	NaN	20,701
gi|470395029|ref|XP_004335030.1|	Ribosomal family s4e, putative	27.273	9.1	2	NaN	20,60729	20,35348
gi|470391709|ref|XP_004334702.1|	Hras1 protein	22.754	5.4	1	NaN	20,50485	20,37581
gi|470431682|ref|XP_004338423.1|	Calponin domain-containing protein	19.77	13.4	2	20,20347	20,38574	20,54161
gi|470417075|ref|XP_004336955.1|	Translation elongation factor Tu, putative	49.291	11.4	5	19,84011	21,41761	19,74516
gi|470522464|ref|XP_004354032.1|	Succinate dehydrogenase and fumarate reductase ironsulfur protein	32.747	4.1	1	20,46832	20,19508	20,32476
gi|470380149|ref|XP_004333661.1|	Actin bundling protein	31.046	12.6	3	NaN	20,3005	20,35434
gi|440790682|gb|ELR11962.1|	Extracellular response kinase, putative	47.967	9.6	4	19,95035	20,0654	20,56151
gi|470486273|ref|XP_004344589.1|	Ras-like protein 1, putative	22.559	12.7	2	NaN	20,19135	20,12326
gi|470380314|ref|XP_004333676.1|	Proteasome subunit alpha type 6, putative	27.045	14.9	3	NaN	20,2876	19,99755
gi|470475061|ref|XP_004342123.1|	GTP-binding protein	24.385	16.3	3	20,20431	20,17913	19,89812
gi|470449066|ref|XP_004340229.1|	Ribosomal protein L6e, putative	27.47	6.1	1	20,014	19,88247	20,32729
gi|470457495|ref|XP_004341141.1|	Calcium-binding mitochondrial carrier protein	80.548	3.4	2	NaN	19,93603	20,19352
gi|470501765|ref|XP_004347187.1|	Microtubule-associated protein (MAP65/ASE1 family)	61.808	2	1	19,9894	19,79853	19,63835
gi|470422827|ref|XP_004337531.1|	Uncharacterized protein ACA1_163460	24.655	10.6	2	NaN	19,79066	19,70578
gi|470510548|ref|XP_004351544.1|	Phosphatidate cytidylyltransferase	39.059	5.1	1	NaN	19,53448	19,76374
gi|470374283|ref|XP_004333084.1|	AcylCoA dehydrogenase, putative	58.05	6.1	3	NaN	19,58344	19,68368
gi|470441553|ref|XP_004339426.1|	Ribosomal protein L10, putative	23.766	10.1	2	19,31586	NaN	19,6768
gi|470482928|ref|XP_004343658.1|	Uncharacterized protein ACA1_099250	33.006	6.2	2	18,84607	18,81541	19,16226

### Size variation and genomic distribution of proteins in cedratvirus pambiensis

**Fig 2:**
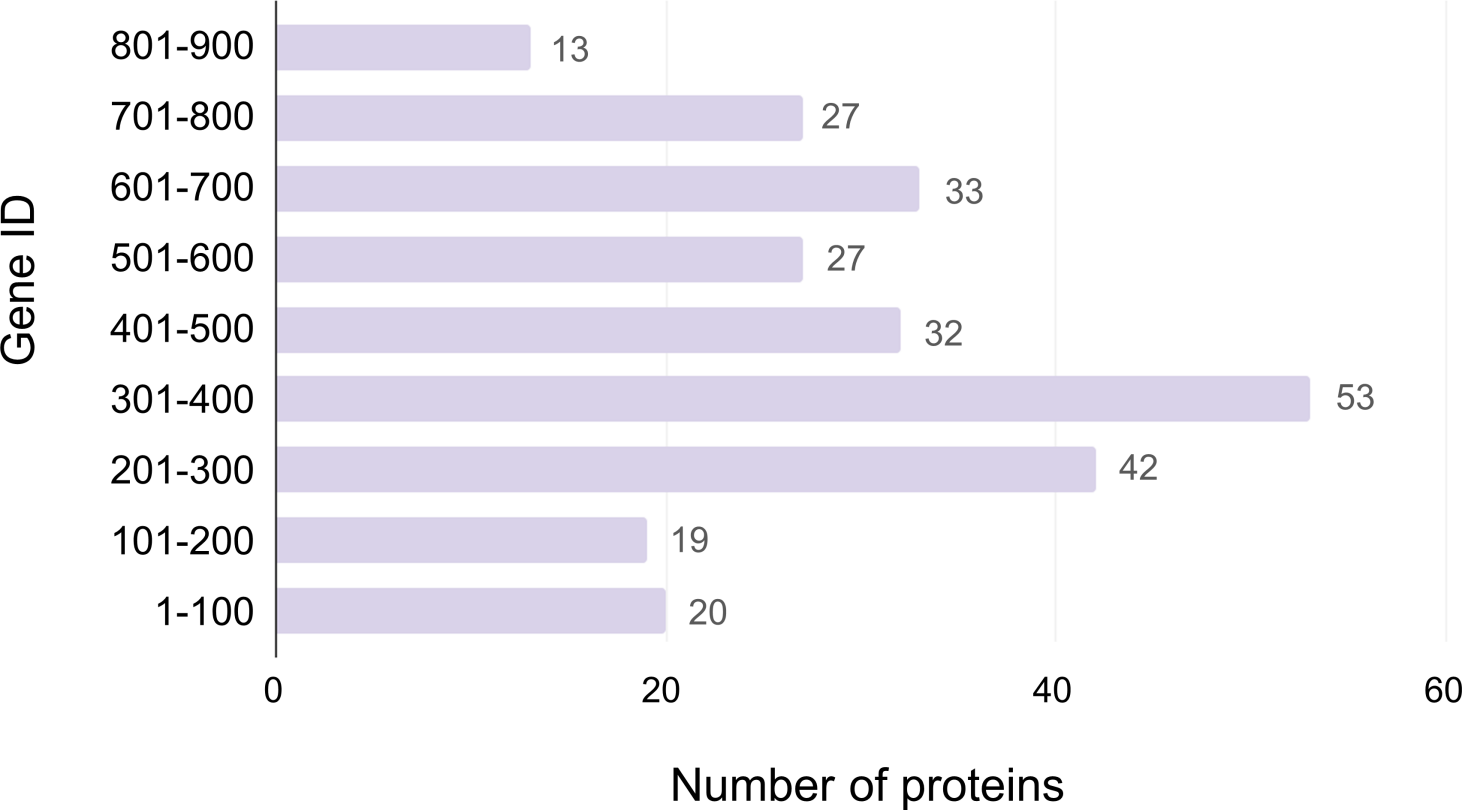
Distribution of the number of proteins in each gene ID range.

The protein sizes identified in this study ranged from 52 to 1,225 amino acids, corresponding to 6 to 135 kDa in mass, with the majority of proteins falling within the 101–300 amino acid range (11–33 kDa). Interestingly, no proteins were identified in the particles within the amino acid positions of 751–950, 1001–1050, and 1101–1200, which may reflect a limitation of the technique, as larger proteins have more difficulty entering SDS-PAGE, or it may be the case that certain protein sizes may be underrepresented or absent in the viral proteome (Fig. 1b). Further analysis revealed that most proteins found in the particles were associated with gene IDs in the 301–400 range (53 genes), followed by the 201–300 range (42 genes). In contrast, gene IDs in the 901–1000 range exhibited a lower number of identified proteins (Fig. 2).

**Fig 3:**
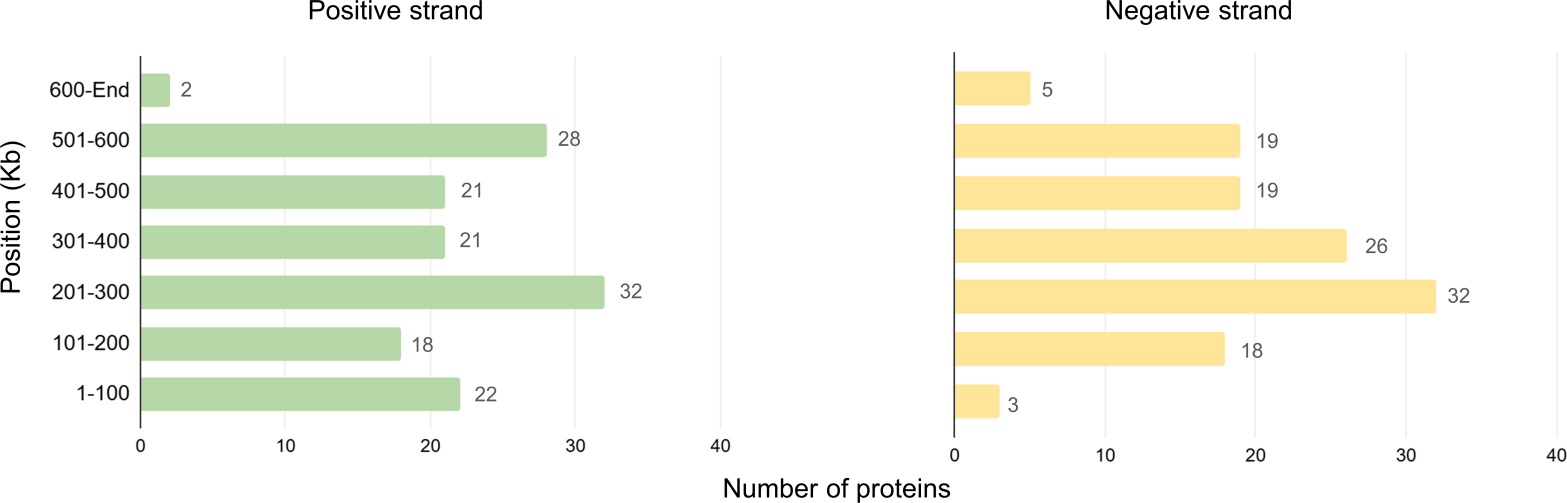
Distribution of genes within the genome, according to their orientation on the positive or negative strands.

In addition to protein size and distribution, we analyzed the particle proteome in terms of the orientation of genes on the positive and negative strands of the genome. The results showed a slightly higher number of proteins encoded by the positive strand (*n* = 144) compared to the negative strand (*n* = 122). When examining the distribution of proteins across different genomic intervals, we found that the distribution was largely similar between the two strands. However, in the 1–100 kb region, the positive strand encoded a higher number of proteins when compared to the same region in the negative strand. The 201–300 kb region is responsible for encoding the largest number of proteins in the particle, suggesting that this region may be a hotspot for transcription or translation activity during the late times of the cycle (Fig. 3). This uneven genomic distribution of virion-encoded genes has been previously observed in proteomic analyses of Pandoravirus, and our findings for cedratvirus are consistent with this trend.

### Comparative proteomic analysis between cedratvirus pambiensis and pithovirus sibericum

**Fig 4:**
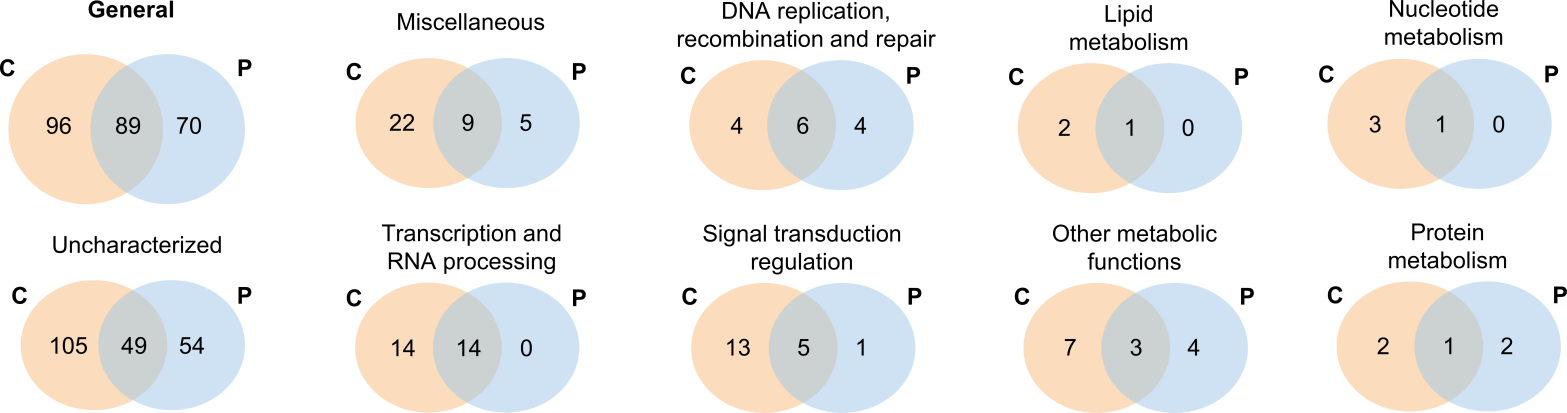
Comparison between cedratvirus pambiensis and pithovirus sibericum proteomes. Eight proteins were not represented because they are only present in the cedratvirus proteome. C: cedratvirus pambiensis. P: pithovirus sibericum.

Among the viruses clustered with cedratvirus pambiensis, only pithovirus sibericum has published proteomic data, identifying 159 proteins (4). Comparative analysis of the two proteomes revealed 89 shared proteins, predominantly uncharacterized (49 proteins, hypothetical proteins included). Other shared proteins were involved in transcription and RNA processing (14 proteins) and DNA replication, recombination, and repair (6 proteins). Additional shared functional categories included signal transduction regulation (5 proteins), miscellaneous functions (9 proteins), lipid metabolism (1 protein), and other metabolic processes (3 proteins) (Fig. 4).

Pithoviruses contain a single protein related to lipid metabolism and another related to nucleotide metabolism, which are shared with cedratvirus. All of the proteins involved in transcription and RNA processing identified in the pithovirus proteome are shared with cedratvirus pambiensis (Fig. 4). Notably, cedratvirus pambiensis possesses unique proteins involved in carbohydrate metabolism (2), host-virus interactions (2), virion structure and morphogenesis (2), and translation (1), which were not identified in the pithovirus proteome.

We performed BLASTp of all cedratvirus pambienis proteins against pithovirus sibericum proteins and observed that 96 (36.1%) did not have any significant similarity, that is, they are exclusive to cedratvitus (Table S2).

We selected the 20 most abundant proteins in the cedratvirus pambiensis proteome, of which 13 are hypothetical proteins, 4 are uncharacterized, 1 HMG box domain-containing protein, 1 NTF2-like domain-containing protein, and 1 protein containing a kinase domain. BLAST analysis of these proteins against the pithovirus sibericum proteome showed no significant similarity for 6 of them (Table 3). The same procedure applied to the 20 most abundant proteins in the pithovirus proteome, which include 17 hypothetical proteins, 1 DNA-binding ferritin-like protein, 1 DNA-directed RNA polymerase RPB10, and 1 transcription elongation factor TFIIS, revealed that 5 proteins had no significant similarity in the cedratvirus proteome. Additionally, for the proteins pv_6, pv_411, pv_114, and pv_456, more than one corresponding match was identified in the cedratvirus genome (Table 3).

**TABLE 3:** Comparison between the most abundant proteins in the cedratvirus pambiensis and pithovirus sibericum proteomes and their orthologs

Position	Cedratvirus protein ID	Ortholog in pithovirus ID	Position	Pithovirus protein ID	Ortholog in cedratvirus ID
1	gene_665 [hypothetical protein]	pv_449 [hypothetical protein]	1	pv_449 [hypothetical protein]	gene_665 [hypothetical protein]
2	gene_621 [hypothetical protein]	pv_46 [hypothetical protein]	2	pv_461 [hypothetical protein]	gene_621 [hypothetical protein]
3	gene_563 [hypothetical protein]	pv_79 [hypothetical protein]	3	pv_93 [hypothetical protein]	No significant similarity found
4	gene_203 [hypothetical protein]	pv_106 [hypothetical protein]	4	pv_106 [hypothetical protein]	gene_203 [hypothetical protein]
5	gene_651 [HMG box domain-containing protein]	pv_449 [hypothetical protein]	5	pv_365 [DNA-binding ferritin-like protein]	No significant similarity found
6	gene_550 [hypothetical protein]	pv_423 [hypothetical protein]	6	pv_6 [hypothetical protein]	gene_608; gene_ 602 [hypothetical protein]
7	gene_450 [uncharacterized protein]	pv_45 [hypothetical protein]	7	pv_100 [hypothetical protein]	No significant similarity found
8	gene_608 [hypothetical protein]	pv_6 [hypothetical protein]	8	pv_384 [hypothetical protein]	gene_157 [NTF2-like domain-containing protein]
9	gene_157 [NTF2-like domain-containing protein]	pv_384 [hypothetical protein]	9	pv_45 [hypothetical protein]	gene_450 [uncharacterized protein]
10	gene_183 [uncharacterized protein]	pv_383 [hypothetical protein]	10	pv_265 [hypothetical protein]	gene_176 [hypothetical protein]
11	gene_325 [uncharacterized protein]	No significant similarity found	11	pv_284 [hypothetical protein]	gene_798 [uncharacterized protein]
12	gene_319 [protein containing kinase domain]	pv_418 [protein kinase]	12	pv_160 [hypothetical protein]	No significant similarity found
13	gene_597 [hypothetical protein]	No significant similarity found	13	pv_1 [hypothetical protein]	No significant similarity found
14	gene_602 [hypothetical protein]	pv_6 [hypothetical protein]	14	pv_256 [hypothetical protein]	gene_176 [hypothetical protein]
15	gene_275 [hypothetical protein]	No significant similarity found	15	pv_383 [hypothetical protein]	gene_183 [uncharacterized protein]
16	gene_415 [uncharacterized protein]	No significant similarity found	16	pv_411 [hypothetical protein]	gene_295; gene_306 [hypothetical protein]
17	gene_237 [hypothetical protein]	pv_106 [hypothetical protein]	17	pv_448 [hypothetical protein]	gene_345 [uncharacterized protein]
18	gene_282 [hypothetical protein]	No significant similarity found	18	pv_114 [DNA-directed RNA polymerase RPB10]	gene_573 [DNA-directed RNA polymerase RPB10]; gene_810 [uncharacterized protein]
19	gene_284 [hypothetical protein]	pv_92 [hypothetical protein]	19	pv_29 [transcription elongation factor TFIIS]	gene_414 [TFIIS transcription elongation factor]
20	gene_479 [hypothetical protein]	No significant similarity found	20	pv_456 [hypothetical protein]	gene_363; gene_372; gene_624 [transmembrane domain-containing protein]

These data demonstrate that the proteomes of cedratvirus pambiensis and ithovirus sibericum have significant differences between them. However, key features are conserved, including a nearly complete set of genes involved in transcription.

## DISCUSSION

Since the discovery of APMV in 2003 (1), giant viruses have been isolated worldwide. They stand out due to their large particle sizes and the complexity of their genomes (3, 4, 7, 12, 16, 18). Despite the growing interest in these viruses, many aspects of their life cycle and interaction with the host or the environment still remain a mystery. Proteomic studies of particles published to date have identified the presence of essential proteins that contribute to the partial independence of giant viruses from their hosts, such as proteins involved in transcription and redox control (2, 3, 12–15). One intriguing feature of giant viruses is the presence of ORFans in their genomes, which are genes with unknown functions but are often identified in proteomic analysis. Proteomic studies may help to elucidate the roles and importance of these proteins in viral biology, manipulation of the host environment, and viral evolution.

The proteomic analysis of cedratvirus pambiensis identified 266 viral proteins and 172 proteins associated with the host *Acanthamoeba castellanii*. The presence of proteins involved in transcription and RNA processing suggests that these viruses may be capable of initiating transcription with a certain degree of autonomy soon after infecting their host. Various metabolism-associated proteins were identified, such as those related to lipid, nucleotide, and carbohydrate metabolism, suggesting potential manipulation of the host environment to benefit viral propagation. These findings suggest that cedratvirus pambiensis virions possess a sophisticated set of proteins that likely play a critical role in initiating and maintaining the viral life cycle within the host amoeba.

Structural proteins were also identified, and two MCP genes were detected, highlighting their essential roles in virion assembly. However, unlike what is described for other nucleocytoviruses as mimivirus and phycodnaviruses, the cedratvirus MCP is not among the most abundant proteins found in the capsid. This suggests that cedratvirus MCP proteins may not serve the same role as they do in other nucleocytoviruses. This is compatible with the cedratvirus non-ichosaedrical, oval-shaped particle. In mollivirus, for instance, MCP remains associated with the particle and acts as a scaffold for virion formation, supporting the idea that MCP functions may have diverged among different lineages (19). Furthermore, 172 host-associated proteins were identified, indicating that host proteins may be incorporated into the viral particles during the late step of the cycle.

Comparative analysis of cedratvirus pambiensis and its relative pithovirus sibericum proteomes revealed 89 shared proteins. Most of them are uncharacterized proteins (49 proteins) but were also identified proteins involved in transcription and RNA processing (14 proteins), DNA replication, recombination, and repair (6 proteins), signal transduction regulation (5 proteins), miscellaneous functions (9 proteins), lipid metabolism (1 protein), nucleotide metabolism (1 protein), and other metabolic processes (3 proteins). We also observed 96 (36.1%) proteins that are exclusive to cedratvirus pambiensis proteome, among them proteins involved in carbohydrate metabolism (2), host-virus interactions (2), virion structure and morphogenesis (2), and translation (1). These results represent one more step forward toward the understanding of the core proteome of the oval-shaped pimascoviruses. It seems that pithoviruses and cedratviruses have their cycle entirely in the host cytoplasm, which is compatible with proteomics and electron microscopy findings. The proteomic analysis of Orpheovirus particles will be useful to a better understanding of the core and exclusive proteomics of this group of viruses.

In conclusion, our data demonstrate that cedratvirus pambiensis particles possess a distinct set of proteins, with significant differences compared to the proteome of its relative, ithovirus sibericum. However, key features are conserved, including a nearly complete set of genes involved in transcription, suggesting that this virus can initiate and complete its life cycle without an apparent nuclear phase. Further research on other pimascoviruses is needed to gain a deeper understanding of the essential core set of proteins shared by this virus group.

## MATERIALS AND METHODS

### Production and purification

*Acanthamoeba castellanii* cells were infected with cedratvirus pambiensis particles at an MOI of 0.01 in glass culture flasks (300 cm^2^) with 35 mL of PYG medium and kept at 30°C in a rotary cell oven for 5 days. After complete cell lysis, the contents of the flasks were collected and centrifuged at 145 × *g* for 10 min. After centrifugation, the supernatant was transferred to a sterile beaker, and the pellet was discarded. This supernatant was added to a sucrose cushion (40%) and then centrifuged at 33,000 × *g* for 1 h, between 4°C and 8°C. The final pellet was resuspended in 10 mL of phosphate-buffered saline, and this content was again added to a sucrose cushion (40%) and centrifuged at 33,000 × *g* for 1 h, between 4°C and 8°C. The centrifugation process was performed twice to obtain the purest sample possible.

### Protein preparation

For protein extraction, 100 million viral particles were resuspended in Laemmli buffer (90 mM dithiothreitol [DTT], 2% SDS, 80 mM Tris-HCl pH 6.8, 10% glycerol, and 0.1% bromophenol blue) for protein extraction and solubilization. Proteins were then separated using SDS-PAGE, and electrophoresis was halted as soon as the samples entered the resolving gel. The gel was stained with Coomassie Brilliant Blue (Bio-Rad), and the single band containing all proteins was excised for in-gel digestion. The experiment was performed in triplicate (see Fig. S1 at https://www.giantviruses.com/sup-material-of-papers/sup-material-the-proteomics-of-the-giant-cedratvirus-particles-reveals-unique-and-shared-features-with-pitho-like-viruses). The excised gel band was destained using a solution of 50% methanol and 2.5% acetic acid, followed by protein reduction with 10 mM DTT and alkylation with 50 mM iodoacetamide in the dark for 30 min. The reaction was quenched by adding 10 mM DTT and incubating in the dark for 15 min. Protein digestion was performed using 1 µg of trypsin (Promega, Madison, WI, USA) at 37°C for 16 h. The reaction was stopped by adding 5% formic acid, and the peptides were extracted with 5% formic acid in 50% acetonitrile. Peptides were desalted using the StageTips method with C18 Empore disks (3M, St. Paul, MN, USA). After desalting, samples were dried in a vacuum concentrator and reconstituted in 10 µL of 0.1% formic acid for further analysis. The data presented represent technical replicates. The same viral preparation was aliquoted, lysed independently, separated by SDS-PAGE, and processed separately for mass spectrometry analysis.

### LC/MS-MS

The samples were analyzed by LC-MS/MS on an Orbitrap Exploris 240 mass spectrometer (Thermo Fisher Scientific, USA) connected to the EASY-nLC 1200 system (Proxeon Biosystems, USA) through a Proxeon nanoelectrospray ion source. Peptides were separated by a 2%–40% acetonitrile gradient over 155 min, using 80% acetonitrile in 0.1% formic acid, in a trap Acclaim PepMap 100 nanoViper 2PK C18 (2 cm × ID75 µm, 3 µm particle size, Thermo Scientific) in line with an analytical PepMap RSLC C18 ES 902 column (50 cm × ID75 µm, 2 µm particle size) at a flow rate of 250 nL/min. The nanoelectrospray voltage was set to 1.7 kV, and the source temperature was 275°C. The full-scan MS spectra (m/z 375–1,500) were acquired in the Orbitrap analyzer after accumulation of a target value of 3e6. Resolution in the Orbitrap was set to *r* = 60,000, and the 20 most intense peptide ions with charge states ≥2 were sequentially isolated to a target value of 3e5 and fragmented by high-energy collisional dissociation (normalized collision energy of 27%). The signal threshold for triggering an MS/MS event was set to 1e4. Dynamic exclusion was enabled with an exclusion duration of 20 s and a repeat count of 1. The maximum injection time was 60 ms.

### Data analysis

Proteins were identified using MaxQuant version 2.4.7.0 against concatenated databases from *Acanthamoeba castellani* and genome sequences (37,160 protein sequences, 15,296,385 residues, April 2024) using the Andromeda search engine. Six-frame translation was performed using the six-frame translation tool embedded in MaxQuant. Carbamidomethylation was set as a fixed modification, and N-terminal acetylation and oxidation of methionine were used as variable modifications. A maximum of two trypsin/P missed cleavages, a tolerance of 10 ppm for precursor mass, and a tolerance of 0.02 Da for fragment ions were set for peptide identification. Protein groups were automatically inferred by the Andromeda engine using the parsimony principle. A maximum of 1% FDR, calculated using reverse sequences, was set for both protein and peptide identification. Protein quantification was performed using the LFQ algorithm implemented in MaxQuant software to reflect a normalized protein quantity deduced from razor + unique peptide intensity values. A minimal ratio count of one was set. Protein identifications assigned as “Reverse,” only identified by “site” and those identified in only one of the samples were excluded from further analysis (see Fig. S2 at https://www.giantviruses.com/sup-material-of-papers/sup-material-the-proteomics-of-the-giant-cedratvirus-particles-reveals-unique-and-shared-features-with-pitho-like-viruses). LFQ intensities were log2-transformed in Perseus version 2.0.11 and used in subsequent analyses. Proteins with one or two peptides and coverage <10%, simultaneously, were removed from the analysis.

### Bioinformatic analyses

The functional annotation of cedratvirus pambiensis proteins was performed using BLASTp against the NCBI NR database under default parameters. The functional categories utilized in this study for both viruses were based on those described by Yutin et al. (2). Comparative analysis of the viral proteomes was performed using BLASTp, aligning all cedratvirus pambiensis proteins against the published pithovirus sibericum proteome (4), with an e-value threshold of 0.001. For the comparative analysis, the most recent functional description of each protein was considered. The same analysis was conducted for the 20 most abundant pithovirus sibericum proteins, aligning them against all cedratvirus pambiensis proteins. All graphs were generated using Microsoft Excel and PowerPoint software.

## Data Availability

The proteomics data have been deposited to the ProteomeXchange database under the accession number PXD069162.
